# Enhancing emotional expression in cat-like robots: strategies for utilizing tail movements with human-like gazes

**DOI:** 10.3389/frobt.2024.1399012

**Published:** 2024-07-15

**Authors:** Xinxiang Wang, Zihan Li, Songyang Wang, Yiming Yang, Yibo Peng, Changzeng Fu

**Affiliations:** ^1^ HACI, Sydney Smart Technology College, Northeastern University, Shenyang, China; ^2^ Hebei Key Laboratory of Marine Perception Network and Data Processing, Northeastern University at Qinhuangdao, Qinhuangdao, China; ^3^ IRL, Graduate School of Engineering Science, Osaka University, Suita, Japan

**Keywords:** human-robot interaction, emotional expression, bionic eye, mechanical tail, user experience, emotion recognition, nonverbal communication, cat-like robot

## Abstract

In recent years, there has been a significant growth in research on emotion expression in the field of human-robot interaction. In the process of human-robot interaction, the effect of the robot’s emotional expression determines the user’s experience and acceptance. Gaze is widely accepted as an important media to express emotions in human-human interaction. But it has been found that users have difficulty in effectively recognizing emotions such as happiness and anger expressed by animaloid robots that use eye contact individually. In addition, in real interaction, effective nonverbal expression includes not only eye contact but also physical expression. However, current animaloid social robots consider human-like eyes as the main emotion expression pathway, which results in a dysfunctional robot appearance and behavioral approach, affecting the quality of emotional expression. Based on retaining the effectiveness of eyes for emotional communication, we added a mechanical tail as a physical expression to enhance the robot’s emotional expression in concert with the eyes. The results show that the collaboration between the mechanical tail and the bionic eye enhances emotional expression in all four emotions. Further more, we found that the mechanical tail can enhance the expression of specific emotions with different parameters. The above study is conducive to enhancing the robot’s emotional expression ability in human-robot interaction and improving the user’s interaction experience.

## 1 Introduction

In recent years, research on the relationship between robots and emotions has made remarkable progress (Savery and Weinberg, 2020). Emotional robots are increasingly used in medical care (Ficocelli et al., 2015), companionship (Karim, Lokman, and Redzuan, 2016), education (Hyun, Yoon, and Son, 2010), and other fields. The effect of emotional expression of robots is crucial to the user’s experience and acceptance during human-computer interaction. Facial expression plays a vital role in the expression of emotion. Facial expressions are a valuable source of information about an individual’s age, and emotional state. The eyes, as the core component of the face, are particularly important in conveying key information about an individual’s emotional and mental state (Emery, 2000). They are the main medium of emotional expression and directly affect the user’s understanding and acceptance of the robot’s emotional state (Chakraborty et al., 2021). Research has demonstrated that incorporating human-like eye expressions in emotional robots can substantially enhance the accuracy of emotion perception (Penčić et al., 2022). Nevertheless, there are still constraints in using robot emotion expressions solely based on human-like eyes. One issue with many current emotional robot designs is that they often resemble animals, yet animal emotional expression primarily relies on body movements, particularly tail movements, rather than individual facial expressions. These designs may benefit from reducing the emphasis on imitating human eyes as the primary means of emotional expression, while overlooking the potential of using eyes and other body movements simultaneously. The current methods cause incongruity between the appearance and behavior of robots can be attributed to bias, which affects the quality of emotional expression. To improve emotional expression, it is suggested to assist the eyes with body movements, as per strategy (Takanishi et al., 2000).

Additionally, the use of suitable animal characteristics as auxiliary methods is an essential approach to enhancing the quality of robot emotional expression. According to research conducted by Kühnlenz et al. (2010), changes in animal features and posture significantly impact the emotional expression quality of a robot. The study also verified that animal posture features become an important factor affecting the clarity of emotional expression information. This effect is particularly notable when interacting with animal-shaped robots and contributes significantly to their credibility. This, in turn, affects the user’s likability and acceptance of the robot. Xie, Mitsuhashi, and Torii (2019) confirmed the potential benefits of tail-assisted expression, which can enhance the intensity and diversity of emotional expression. Furthermore, the history of human-animal interaction provides a prototype for the behavioral model of social robot agents (Krueger et al., 2021). For instance, observing the behavior pattern of a cat’s tail is often an important way to understand its emotions. This behavioral model provides a prototype for cat-like robots to express emotions through tail-assisted eyes. It is noteworthy that there are few studies on the emotional expression of tail and eye coordination in the field of human-computer interaction. Therefore, this study aims to design strategies for cooperative emotional expression using tail and eye movements, and to evaluate the impact of different kinematic strategies on emotional expression.

In order to explore how to combine the advantages of human-like eye emotion expression and tail emotion expression in emotional interaction robots, our research has made the following contributions:

• We enhanced the accuracy of the robot’s emotional expression by utilizing the mechanical tail to complement the emotional cues conveyed by the eyes.
• Inspired by the sea snake robot study (Ming et al., 2015), we proposed various emotion expression strategies based on tail movement patterns and eye coordination. We also discussed the effects of three factors: waveform, amplitude, and frequency, on robot emotion expression.
• We invited 720 participants to evaluate the robot’s emotional expression under different conditions and motion modes, and received detailed feedback on the robot’s emotional expression for different emotions. It was found that the frequency and amplitude of tail movement significantly affect the emotional expression, animacy, and user affection of the robot.

## 2 Related works

### 2.1 Emotional expression of the eyes

Whether it is human-human interaction or human-robot interaction, eye contact elicits increased affection and attention related psycho-physiological responses (Kiilavuori et al., 2021). Eyes can indicate mental states and show the purpose of social robots (Fong, Nourbakhsh, and Dautenhahn, 2003). To further understand the rational approach to human-machine eye interaction. Xu, Zhang, and Yu (2016) found that the more often a robot looks at a human user’s face, the more mutual gazing and eye contact between the two occurs by examining at what moment and in what way the robot should look at the human user’s face. In addition, when more eye contact was successfully established and maintained, participants showed more coordinated and synchronized multi-modal behaviors between speech and gaze. It seems that we can make eye contact if we look at each other. However, Miyauchi et al. (2004) argue that this alone cannot complete eye contact. In addition, we need to be aware of being looked at by each other. Considering the two cases, Dai Miyauchi et al. propose a method of active eye contact for human-robot communication considering both conditions. The robot changes its facial expressions according to the observation results of the human to make eye contact. Then, they present a robot that can recognize hand gestures after making eye contact with a human to show the effectiveness of eye contact as a means of controlling communication. Jatmiko, Ginalih, and Darmakusuma (2020) expresses seven different emotions by designing a single-eyed 2D avatar that moves the upper and lower eyelids. Comparing Virtual Agents to physical embodiments, the participants had almost similar perceptions of the eyelids, but there is still a part of the emotional expression that is not easy to distinguish. Although previous research has shown that a portion of the emotions expressed by the eye model can be well recognized by the user, e.g., surprise, disgust, and neutrality, relying on eye expression alone may suffer from inconspicuous emotional expression in happiness and anger.

### 2.2 Emotional expression of the tail

The design of the emotional behavior of humanoid robots has attracted the attention of many scholars. Guo et al. (2019) argue that fear and anger behaviors in humanoid robots require larger and more complex amplitude movements compared to happy, neutral, and sad behaviors. Sato and Yoshikawa (2004) presented computer-morphing animations of the facial expressions of six emotions to 43 subjects and asked them to evaluate the naturalness of the rate of change of each expression. The results showed that the naturalness of the expressions depended on the frequency of change, and the patterns for the four frequencies differed with the emotions. Sowden et al. (2021) believe the kinematics of people’s body movements provide useful cues about emotional states. In addition to frequency, the facial movement kinematics also contribute independently and add further value to emotion recognition. Based on humanoid robots, Singh and Young (2013) presented a dog-tail interface for utility robots and investigated a base case of people’s reactions to the tail, discovering that different parameters of tail motion and shape can affect emotional expression in robots. In their study of emotionally interactive robots to help children with autism in early therapy, Lee et al. (2014) used eye contact between cat-like robots and users and body movements to discover that cat-like robots can be therapeutic through appropriate interactions. Although previous studies have shown that frequency, amplitude, and waveform can affect emotional expression to varying degrees, the collaboration between the mechanical tail and eye expression and the interactions between the three parameters have not been adequately discussed, and it remains to be demonstrated as to the efficacy of applying these parameters to a cat-like emotionally interactive robot.

## 3 Methodology

### 3.1 Cat-like robot system

**FIGURE 1:**
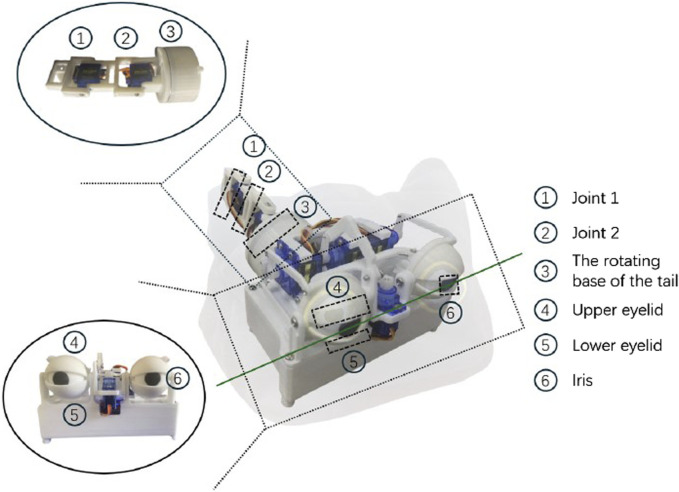
The designed cat-like robot with fur coat. ①②③ demonstrates the hardware structure of mechanical tail; ④⑤⑥ show the hardware of bionic eyes.

This work designs an animaloid robot system with a bionic eyeball (④⑤⑥ in Figure 1), a mechanical tail (①②③ in Figure 1), and a covered fur coat, as shown in Figure 1. Specifically, the bionic eyeball consists of six SG90 servos, an ESP8266 development board, and an MP1584EN 5V buck module designed to allow for eyelid and iris movement. The mechanical tail, on the other hand, consists of three SG90 servos and a Raspberry Pi.

The control system consists of two parts: the mechanical tail and the bionic eyeball, which are initiated to start at the same time. Once the start-up procedure is triggered, the tail and eyeball begin to operate independently, each according to its own motion logic. Their operating cycles are roughly aligned to maintain consistency.

The cat-like robot’s mechanical tail is engineered with a tri-joint mechanism, where the two joints (Figure 1) distal to the base are designed for the movement within a shared plane. These distal joints, referred to as the joint 1 and joint 2, are capable of executing a wide anger of motion. Each joint allows for a full 180-degree rotational range.The base joint (Figure 1) operates in a plane perpendicular to the other two, endowed with the ability to rotate 180°, providing the tail an additional dimension of expressiveness.

**FIGURE 2:**
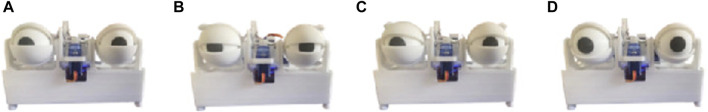
The human-like gazes with four emotions. **(A)** happy; **(B)** sad; **(C)** anger; **(D)** surprise.

The bionic eyeball is designed to mimic an animal’s eye and can express four distinct emotions (Figure 2). The main emotional components of the bionic eyeball are the upper eyelids (Figure 1), lower eyelids (Figure 1) and the iris (Figure 1). The upper eyelid can move in the upper region divided by the green line in Figure 1, while the lower eyelid can move in the lower region. The iris can move freely within a circular area with the center as the origin.

### 3.2 Robot movement implementation

Based on the study of Jatmiko, Ginalih, and Darmakusuma (2020), we replicated four emotional expressions. The expression of each emotion is characterized by:

• **Happiness:** Upturned cheeks, lower eyelids pushed upwards, elevated upper eyelid;
• **Sadness:** A slight squinting of the eyes, a drooping of the upper eyelid;
• **Anger:** Focused enlarged eyes with lower upper eyelids;
• **Surprise:** Wide-open eyes, irises fully visible;

In this system, we use the UDP protocol to transmit commands to wirelessly control the eyelid and eye movements of the eyeball. A variety of common emotional movement patterns are burned on the ESP8266 and an interface for UDP transmission of commands is provided to facilitate control. Meanwhile, we designed two different input functions for the tail, square wave and sine wave, to study the effect of tail motion on the robot’s emotional expression.The sine wave and square wave were chosen because these two waveforms have different frequency characteristics and harmonic distributions, which may induce different effects (Teng, 2011). Sine wave is often used to simulate natural changing trends, such as respiratory behavior or other vital signs (Islam et al., 2022). Due to its continuous and smooth waveform properties, sine wave behaves more naturally in simulating biological processes. In contrast, square wave has more abrupt waveform characteristics and their spectrum mainly contains odd harmonics. This makes square wave more suitable for producing sharp, sudden stimuli (Hunter and Jasper, 1949), whose rapidly changing properties may trigger stronger responses. Sine and square waves have potential roles in simulating biological processes or inducing different effects (Teng, 2011).A change in the input function affects the motion pattern of the tail. When the input is a sine wave, the tail moves at a variable frequency over an angular interval, whereas when the input is a square wave, the tail moves at a constant frequency over an angular interval that stops when it oscillates to its maximum magnitude. The equation for a sine wave is given by (the variables in the equation are shown in Table 1):

$$y=Amp\times \sin\left(1/Freq\times t\right)+1500$$ (1)

The equation for a sine wave is given by (the variables in the equation are shown in Table 1):

$$y=\left\{\begin{array}{cc} Amp\; & t<\left(n+1/2\right)\times 1/Freq\\ -Amp\; & \left(n+1/2\right)\times 1/Freq<t<\left(n+1\right)\times 1/Freq \end{array}\right.$$ (2)

**FIGURE 3:**
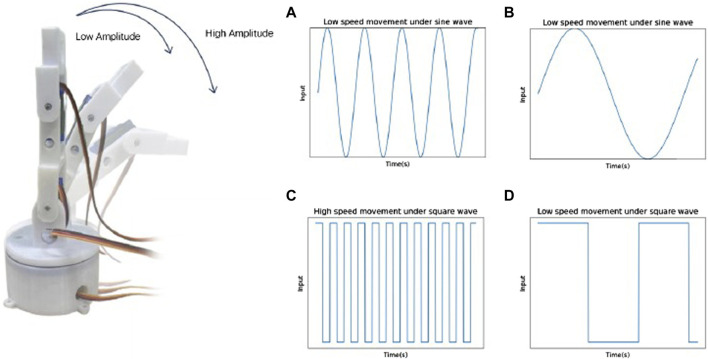
The designed tail trajectories with different amplitude and frequency. **(A)** High frequency movement under sine wave, **(B)** Low frequency movement under sine wave, **(C)** High frequency movement under square wave, **(D)** Low frequency movement under square wave.

By adjusting the amplitude and frequency of the function, it is categorized into four different cases, including high amplitude and high frequency, high amplitude and low frequency, low amplitude and high frequency, and low amplitude and low frequency. The value of the function amplitude affects the maximum amplitude at which the tail can swing. When the tail is input at a high amplitude, the maximum amplitude at which the tail can swing is greater compared to a low amplitude, as shown in Figure 3. On the other hand, frequency affects the rate at which the tail swings.The inputs of high and low frequencies of triangular and square waves are illustrated in Figures 3A–D. In the figure, it can be seen that the function changes faster and more drastically under the condition of high frequency input. Correspondingly, the tail oscillates in a more rapid manner.

**TABLE 1:** Kinematic control system variables.

Name	Definition
Amp	Maximum amplitude of the function
Freq	Frequency of the function
t	Time variable

## 4 Experiment

### 4.1 Experiment 1: assessment of bionic eyeballs’ impact on expressive emotions

#### 4.1.1 Subject

For this study, 360 volunteers (M = 184, F = 176, age = 37.74), and an age distribution between 21 and 54 years old were selected from a variety of backgrounds. These individuals were asked to complete a topic-specific questionnaire to assess how well the bionic eyeball expressed emotions. We gathered the participants’ responses to the four distinct emotions (happiness, sadness, anger, and surprise) that the eyeball robot displayed by using the Mean Opinion Score (MOS) questionnaire (Streijl and Hands, 2016). Furthermore, we evaluated the robot’s Animacy and Likability in terms of various aspects (Vitality, Liveness, Organicity, Realism, Reactivity, Affinity, Likability, Friendliness, Affection, Pleasure, and Quality) using the Godspeed questionnaire (Weiss and Bartneck, 2015). The subsequent chapters will delineate the detailed protocol and expound upon the experimental results.

#### 4.1.2 Condition

The four primary eyeball motions employed in this study were happiness, sadness, anger, and surprise. The kinematic design of the bionic eyeball robot’s motion was derived from the research conducted by Jatmiko, Ginalih, and Darmakusuma (2020), who explored diverse expressive movement designs for the identical model of the bionic eyeball robot. We prepared a 10-s video sample for each emotional condition in this experiment to illustrate the transition in expression, thereby validating the accuracy of emotional expression. An online questionnaire was employed to gather data for this investigation. Following the viewing of a video depicting the movement of a bionic eyeball, participants were instructed to respond to 15 questions. Four of these questions employed MOS to evaluate the robot’s emotional expressions, encompassing dimensions like happiness, sadness, anger, and surprise (Streijl and Hands, 2016). The remaining 11 questions, drawn from the Godspeed questionnaire, were utilized to assess animacy and likability (Weiss and Bartneck, 2015). To ensure precise feedback on the conveyed emotions by the eyeball robot, participants will respond to 15 questions for each of the four emotion-inducing videos in the modified questionnaire. This results in a total of 60 questions per questionnaire.

#### 4.1.3 Procedure

**FIGURE 4:**
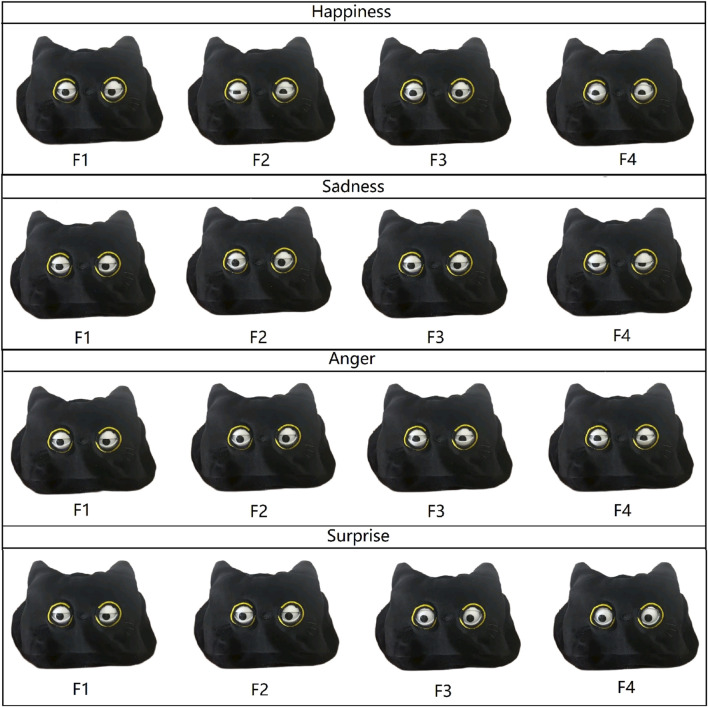
Eyeball movement sequences of a cat-like robot expressing four different emotions: (F1) Eyeball movement at the start; (F2) Eyeball movement at an early intermediate stage; (F3) Eyeball movement at a later intermediate stage; (F4) Eyeball movement at the conclusion.

Before commencing the experiment, participants underwent a detailed briefing outlining the objects and content of this experiment. Additionally, they received instructions on how to complete the MOS questionnaire (Streijl and Hands, 2016). This procedure was implemented to ensure that participants were thoroughly informed about the upcoming experiment and that their participation was entirely voluntary. The experiment received approval from the University Ethics Committee, affirming its alignment with ethical guidelines for the involvement of human subjects in research. The subsequent list outlines the specific steps of the experiment:

1. Manipulation check for emotional gaze design: Initially, a small-scale pilot experiment was conducted to validate the efficacy of the motion design based on Jatmiko [34]. In this phase, twelve participants were selected and organized into three-person groups. They were presented with four 10-s videos illustrating different emotions (happiness, sadness, anger, surprise). Subsequently, participants were required to identify and rate the accuracy of the displayed emotion (happiness, sadness, anger, surprise) by the bionic eyeball in each video.
2. Questionnaire Overview: Upon accessing the online form, participants were initially provided with an explanation of the experiment’s objectives and the questionnaire’s content. This encompassed a comprehensive overview of the emotional scoring content incorporated in the MOS questionnaire (ibid.). Participants were also prompted to furnish basic personal information, such as age and gender.
3. Video Presentation: The participants viewed four 10-s video segments, each demonstrating the eye movements of a cat-like bionic eyeball robot under four different emotional states. Figure 4 displays the process of the movements.
4. Completing the Emotion Assessment (Streijl, Winkler, and Hands, 2016) and Godspeed Questionnaire (Weiss and Bartneck, 2015): Following the video, participants were required to assess each of the four emotions depicted in the film using distinct emotional scales. To prevent participants from discerning the intended emotional cues from the questionnaire items, each emotional video was accompanied by ratings for all four emotions. However, only the scores corresponding to the emotion depicted in each specific video were utilized in the actual data analysis. This method ensured that participants’ assessments were unbiased by their expectations or assumptions about the video content. The Likert scale ranged from “Neutral” to “Happy,” “Neutral” to “Sad,” “Neutral” to “Angry,” and “Neutral” to “Surprise,” with five possible responses. Additionally, to obtain a comprehensive insight into participants’ perceptions of the robot, the questionnaire included evaluation queries regarding the robot’s animacy and likability. These encompassed six aspects of animacy (vitality, organicity, realism, responsiveness, and affinity) and five aspects of likability (likability, friendliness, pleasantness, and overall quality), respectively. A five-point Likert scale was utilized for scoring, with response options ranging from “Strongly Disagree” to “Strongly Agree” for each question.

The hypotheses for Experiment 2 will be examined using the data acquired through the aforementioned procedure.

### 4.2 Experiment 2: evaluation of the emotional expression effect of the combination of bionic eyeballs and mechanical tails

#### 4.2.1 Condition

**FIGURE 5:**
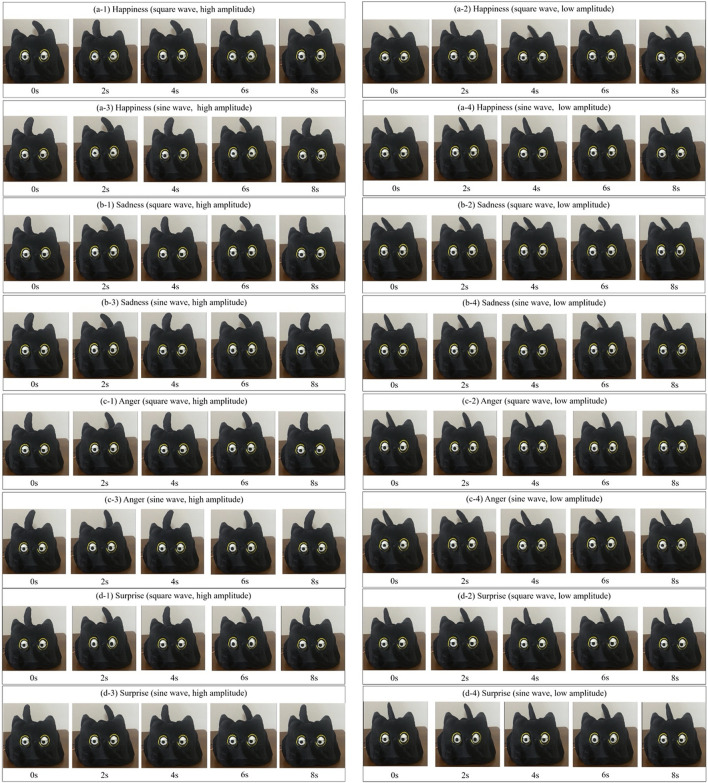
The conditions of the robot that combines bionic eyeballs and mechanical tail. Only waveforms and amplitudes are discussed here. Five video frames were captured for each condition. (a-1) Square wave, high amplitude movement; (a-2) Square wave, low amplitude movement; (a-3) Sine wave, high amplitude movement; (a-4) Sine wave, low amplitude movement; (b-1) Square wave, high amplitude movement; (b-2) Square wave, low amplitude movement; (b-3) Sine wave, high amplitude movement; (b-4) Sine wave, low amplitude movement; (c-1) Square wave, high amplitude movement; (c-2) Square wave, low amplitude movement; (c-3) Sine wave, high amplitude movement; (c-4) Sine wave, low amplitude movement; (d-1) Square wave, high amplitude movement; (d-2) Square wave, low amplitude movement; (d-3) Sine wave, high amplitude movement; (d-4) Sine wave, low amplitude movement.

In this experiment, we explore how to achieve richer and more delicate emotional expression for robots by adding mechanical tails based on the bionic eyeballs. The design of the mechanical tails takes into account three key independent variables: the frequency of movement of the mechanical tails (high and low), the amplitude of movement of the mechanical tails (high and low), and the waveforms (square wave and sine wave). Different combinations of these variables can create tail movements with different characteristics, simulating different emotional expressions. Under each experimental condition, the robot was required to express four basic emotions: Happiness, sadness, anger, and surprise. The combination of waveform and amplitude is represented by Figure 5.

#### 4.2.2 Subject

A total of 360 experimental participants aged 22 to 54 were recruited from a variety of backgrounds to ensure that the findings were broadly representative. These participants were invited to work in groups to evaluate the emotional expression effect under the combination of mechanical tails and bionic eyeballs. The experiment is divided into eight groups, classified according to the movement frequency, movement amplitude and input waveform of the mechanical tails. The specific participant groups are as follows: Group 1: 45 participants (M = 26, F = 19, age = 39.28) evaluated the robot’s expression of four emotions under high amplitude, high frequency, and square wave conditions; Group 2: 45 participants (M = 19, F = 26, age = 37.4) evaluated the robot’s expression of four emotions under high amplitude, low frequency, square wave conditions; Group 3: 45 participants (M = 22, F = 23, age = 36.9) evaluated the robot’s expression of four emotions under low amplitude, high frequency, square wave conditions; Group 4: 45 participants (M = 21, F = 24, age = 38.12) evaluated the robot’s expression of four emotions under low amplitude, low frequency, and square wave conditions; Group 5: 45 participants (M = 23, F = 22, age = 34.7) evaluated the robot’s expression of four emotions under high amplitude, high frequency, and sine wave conditions; Group 6: 45 participants (M = 25, F = 20, age = 35.3) evaluated the robot’s expression of four emotions under high amplitude, low frequency, sine wave conditions; Group 7: 45 participants (M = 26, F = 19, age = 35.9) evaluated the robot’s expression of four emotions under low amplitude, high frequency, sine wave conditions; Group 8 : 45 participants (M = 22, F = 23, age = 36.04) evaluated the robot’s expression of four emotions under low amplitude, low frequency, and sine wave conditions. Throughout the study, in order to maintain the objectivity and fairness of the experiment, participants were not informed of the design and results of the other groups.

#### 4.2.3 Procedure

Before the experiment began, the participants received a series of detailed introductions, including the purpose, content, and process of the experiment, to ensure that each participant had a full understanding of the experiment they were about to participate in. In order to protect the rights of the participants and the ethics of the experiment, this experiment has been reviewed and approved by the school ethics committee and meets the ethical requirements for human participation in research. The specific experimental steps are as follows:

1. Participants first read the same questionnaire instructions as in Experiment 1 to understand the purpose of the experiment and the general content of the questionnaire. At the same time, participants also need to submit their basic information, such as age and gender.
2. After ensuring that the participants fully understood the basic information of the experiment, they were asked to watch one of the sets of videos. These videos show robots using a combination of bionic eyeballs and mechanical tails to express different emotions. Each video was designed to be as standardized and consistent as possible to ensure that each subject received the same emotional stimulus. Participants were unaware of the emotion shown in the video before watching it, as they were not informed about the content in advance. They needed to make judgments based solely on their own perceptions to assess their natural responses to the video.
3. Immediately after watching the video, each participant needs to use the MOS questionnaire to rate their emotional experience (Streijl, Winkler, and Hands, 2016). In order to accurately measure the emotional response of the participants, in addition to the emotional dimension, this experiment also specifically selected the two dimensions of animacy and likability to deeply explore the complexity of the robot’s emotional expression.

In addition, the eight groups of questions in this experiment were set the same. The purpose was to evaluate the impact of adding mechanical tails on the intensity of the robot’s emotional expression and the impact of the mechanical tails on the robot’s emotional expression under different combinations of conditions. In this way, researchers can compare and analyze data under different experimental conditions more effectively, thereby drawing more accurate and comprehensive research conclusions.

#### 4.2.4 Hypothesis

The hypotheses of this study are as follows:

• **H1:** Compared with using bionic eyeballs alone, the combination of mechanical tails and bionic eyeballs will improve the accuracy, animacy, and likability of emotional expression;
• **H2:** Mechanical tails’ amplitude significantly affects the expression of emotions. High amplitude is better at conveying happiness and surprise emotions, while low amplitude is better at conveying sadness and anger emotions;
• **H3:** Mechanical tails’ frequency significantly affects the expression of emotions. High frequency is better at conveying happiness and surprise emotions, while low frequency is better at conveying sadness and anger emotions;
• **H4:** Mechanical tails’ waveform significantly affects the expression of emotions. Square waves are better at conveying happiness and surprise emotions, while sine waves are better at conveying sadness and anger emotions;
• **H5:** Individual amplitude factors, frequency factors, and waveform factors significantly affect the animacy score. Interactions between factors did not significantly affect animacy scores. Among them, high amplitude, high frequency, and square waves better express animacy;
• **H6:** Individual amplitude factors, frequency factors, and waveform factors significantly affect the likability score. Interactions between factors did not significantly affect likability scores. Among them, low amplitude, low frequency, and sine waves better express likability;
• **H7:** The interaction of frequency, amplitude, and waveform significantly enhances the expression of emotions, showing stronger animacy and likability;

## 5 Result

### 5.1 Comparing emotional expression impact: bionic eyeball alone vs. bionic eyeball with mechanical tail

In the brief preliminary experiment of Experiment 1, all twelve participants accurately identified the corresponding emotions, validating the precise emotional expression by the ocular robot. Building on this, the study conducted a comparison of emotion scores in two scenarios: with and without the mechanical tail. An independent sample *t*-test was employed to investigate the impact of the mechanical tail on the robot’s ability to convey emotion. In the research design, four distinct groups were delineated as subgroups (each subgroup further divided into two subgroups with and without the tail), resulting in four independent sample t-tests to yield diverse test outcomes. From these subgroups, the study extracted the emotion scores (Happiness, Sadness, Anger, and Surprise) and the specific differences between the corresponding Animacy Score and Likability Score for each emotion, both with and without the addition of the mechanical tail. The following eight groups were created based on the questionnaire results:

• **G1:** No Tail - Happiness, Animacy, and Likability Scores under Happiness Emotion.
•**G2:** With Tail - Happiness, Animacy, and Likability Scores under Happiness Emotion.
•**G3:** No Tail - Sadness, Animacy, and Likability Scores under Sadness Emotion.
• **G4: With** Tail - Sadness, Animacy, and Likability Scores under Sadness Emotion.
• **G5: No** Tail - Anger, Animacy, and Likability Scores under Anger Emotion.
• **G6:** With Tail - Anger, Animacy, and Likability Scores under Anger Emotion.
• **G7:** No Tail - Surprise, Animacy, and Likability Scores under Surprise Emotion.
• **G8:** With Tail - Surprise, Animacy, and Likability Scores under Surprise Emotion.

The tail-free groups served as control groups in the independent samples *t*-test analysis. The results revealed significant differences, at the 0.01 significance level, between the three robot scores with the added mechanical tail and the three robot scores of the control group for each of the four emotions. Moreover, a mean comparison indicated that the robots equipped with mechanical tails scored higher than those without mechanical tails for each of the four emotional expressions, as illustrated in Table 2. In each emotional video segment, ratings for three additional emotions were included to ensure that participants could not deduce the intended emotional setting from the questionnaire design, thereby minimizing potential response biases. An independent samples *t*-test was employed to evaluate the impact of the mechanical tail on the robot’s ability to convey emotions accurately. As a result, the table selectively displays only the *t*-test results that are relevant to the specific emotions depicted.

**TABLE 2:** *t*-test results were employed to compare scores across three types, assessing variations in emotions (happiness, sadness, anger, and surprise) with and without a mechanical tail (w/tail and w/o tail), along with Mean, t-statistic, *p*-values, and Cohen’s d. The analysis included four emotional states and GodSpeed scores (Animacy and Likability) (Weiss and Bartneck, 2015).

	Variable	Condition	Mean	t	p	d
**Happiness set**	Happiness Score	w/tail	4.114	20.276	**0.000**	1.511
		w/o tail	2.411			
	Animacy Score	w/tail	3.525	14.769	**0.000**	1.101
		w/o tail	2.324			
	Likability Score	w/tail	3.530	14.529	**0.000**	1.083
		w/o tail	2.344			
**Sadness set**	Sadness Score	w/tail	3.969	18.564	**0.000**	1.384
		w/o tail	2.342			
	Animacy Score	w/tail	3.510	14.700	**0.000**	1.101
		w/o tail	2.324			
	Likability Score	w/tail	3.503	13.973	**0.000**	1.083
		w/o tail	2.344			
**Anger set**	Anger Score	w/tail	3.961	16.710	**0.000**	1.245
		w/o tail	2.403			
	Animacy Score	w/tail	3.531	15.052	**0.000**	1.122
		w/o tail	2.324			
	Likability Score	w/tail	3.526	14.414	**0.000**	1.074
		w/o tail	2.344			
**Surprise set**	Surprise Score	w/tail	3.762	15.564	**0.000**	1.160
		w/o tail	2.317			
	Animacy Score	w/tail	3.524	14.725	**0.000**	1.098
		w/o tail	2.324			
	Likability Score	w/tail	3.497	14.241	**0.000**	1.061
		w/o tail	2.344			

Bold *p*-values indicate values less than 0.05, and values that are both bold and underlined indicate *p*-values less than 0.01.

Based on the *t*-test results outlined in Table 2, the comparison of scores between participants with and without the mechanical tail across four emotional contexts yielded significant outcomes.

In the happiness emotion, the group with tails, consisting of 360 participants (M = 4.114, SD = 0.948), exhibited significantly higher scores indicating the robot’s display of happiness compared to the control group of 360 participants (M = 2.411, SD = 2.411), as denoted by a marked difference (t (54.734) = 20.276, *p* < 0.001, d = 1.511). This implies a substantial enhancement in positive responses to the robot’s expression of happiness attributed to the presence of the mechanical tail. Similarly, in animacy scores, the tail group (M = 3.525, SD = 1.002) outperformed the control group (M = 2.411, SD = 2.324), resulting in a significant difference (t (22.349) = 14.769, *p* < 0.001, d = 1.101). In likability scores, the tail group (M = 3.530, SD = 1.018) surpassed the control group (M = 2.344, SD = 1.168), exhibiting a significant difference (t (11.305) = 14.529, *p* = 0.001, d = 1.083). Furthermore, the smaller standard deviations of all three scores in the tail group (SD = 0.948, 1.002, 1.018) compared to the control group (SD = 2.411, 2.324, 1.168) indicate a more centralized and consistent response pattern in the tail group. This suggests that the mechanical tail provided an effective and unified framework for interpreting the robot’s expression of happy emotions.

In the sadness emotion, the group with tails, consisting of 360 participants (M = 3.969, SD = 1.075), exhibited significantly higher scores for sadness in response to the robot displaying happy emotions compared to the control group with 360 participants (M = 2.342, SD = 1.270), showing a significant difference (t (23.029) = 18.564, *p* < 0.001, d = 1.384). This outcome indicates a substantial improvement in positive responses to the robot’s expression of sad emotions attributed to the presence of the mechanical tail. Similarly, in animacy scores, the tail group (M = 3.510, SD = 0.984) scored higher than the control group (M = 2.324, SD = 1.173), showing a significant difference (t (21.097) = 14.700, *p* < 0.001, d = 1.096). In likability scores, the tail group (M = 3.503, SD = 1.055) surpassed the control group (M = 2.344, SD = 1.168), showing a significant difference (t (5.278) = 13.973, *p* < 0.001, d = 1.041). Similar to the results in the happy emotion context, the smaller standard deviations of all three scores for participants in the tail group (SD = 1.075, 0.984, 1.055) compared to the control group (SD = 1.270, 1.173, 1.168) suggest more focused and consistent scores in the tail group. This implies that the mechanical tail provides a more accurate and consistent expression of sad emotions for the robot.

In the anger emotion, the group with tails, comprising 360 participants (M = 3.961, SD = 1.063), demonstrated significantly higher anger scores in response to the robot displaying happy emotions compared to the control group of 360 participants (M = 2.403, SD = 1.415), as indicated by a substantial difference (t (33.953) = 16.710, *p* < 0.001, d = 1.245). This outcome points to a noteworthy improvement in positive responses to the robot’s representation of angry emotions facilitated by the presence of the mechanical tail. Similarly, in animacy scores, the tail group (M = 3.531, SD = 0.969) scored higher than the control group (M = 2.324, SD = 1.173), resulting in a significant difference (t (24.748) = 15.052, *p* < 0.001, d = 1.122). In likability scores, the tail group (M = 3.526, SD = 1.028) exceeded the control group (M = 2.344, SD = 1.168), exhibiting a significant difference (t (9.234) = 14.414, *p* < 0.001, d = 1.074). Analogous to the findings under Happy Emotions, the reduced standard deviations of all three scores for participants in the tail group (SD = 1.063, 0.969, 1.028) compared to the control group (SD = 1.415, 1.173, 1.168) indicate more focused and consistent scores in the tail group, suggesting that the mechanical tail provides a more accurate and consistent expression of angry emotions for the robot.

In the surprise emotion, the group with tails, consisting of 360 participants (M = 3.762, SD = 1.184), selected significantly higher surprise scores for the robot displaying happy emotions compared to the control group of 360 participants (M = 2.317, SD = 1.306), as evidenced by a significant difference (t (23.029) = 15.564, *p* < 0.001, d = 1.160). This result indicates a substantial enhancement in positive responses to the robot’s expression of surprised emotions attributed to the presence of the mechanical tail. Similarly, in animacy scores, the tail group (M = 3.524, SD = 1.008) scored higher than the control group (M = 2.324, SD = 1.173), resulting in a significant difference (t (21.097) = 14.725, *p* < 0.001, d = 1.098). In likability scores, the tail group (M = 3.497, SD = 0.999) outperformed the control group (M = 2.344, SD = 1.168), exhibiting a significant difference (t (5.278) = 14.241, *p* < 0.001, d = 1.061). Similar to the results in the happy mood context, the reduced standard deviations of all three scores for participants in the tail group (SD = 3.762, 1.008, 0.999) compared to the control group (SD = 1.306, 1.173, 1.168) suggest more focused and consistent scores in the tail group. This implies that the mechanical tail provided a more accurate and consistent expression of both the robot’s surprised emotions.

### 5.2 The auxiliary effects of different mechanical tails’ combinations on emotional expression

The study used the three-way ANOVA and the *post hoc* test to evaluate the emotional score of the robot after adding the mechanical tails. The changes in the combination of the mechanical tails affect the robot’s emotional score, including the emotional scores of happiness, sadness, anger, and surprise, and the animacy score and likability score corresponding to each emotion’s specific impact. Using the amplitude, frequency, and waveform of the mechanical tails as independent variables, three-way ANOVA analyses and *post hoc* test comparisons were conducted to obtain significant results. The relevant detailed results are shown in Table 3, Table 4, Table 5, Table 6, Table 7 and Table 8.

**TABLE 3:** Statistical results for the 3-way ANOVA under happiness, sadness, anger, and surprise emotions. The results display scores for emotional intensity, along with Mean-square, F-values and *p*-values. A represents amplitude, F represents frequency, and W represents waveform.

		Happiness score			Sadness score			Anger score			Surprise score		
		MS	F	p	MS	F	p	MS	F	p	MS	F	p
**Happiness Set**	A	4.669	5.306	**0.022**	0.000	0.000	1.000	1.111	0.583	0.446	0.100	0.046	0.830
	F	1.736	1.973	0.161	7.511	3.511	0.062	2.844	1.492	0.223	0.044	0.021	0.886
	W	0.225	0.256	0.613	5.878	2.748	0.098	1.344	0.705	0.402	0.044	0.021	0.886
	A * F	5.625	6.392	**0.012**	0.100	0.047	0.829	3.600	1.888	0.170	1.111	0.516	0.473
	A * W	0.003	0.003	0.955	0.711	0.332	0.565	2.500	1.311	0.253	0.044	0.021	0.886
	F * W	0.069	0.079	0.779	0.711	0.332	0.565	0.011	0.006	0.939	0.278	0.129	0.720
	A * F * W	0.225	0.256	0.613	0.278	0.130	0.719	0.011	0.006	0.939	0.100	0.046	0.830
**Sadness set**	A	0.336	0.153	0.696	5.625	5.335	**0.021**	0.100	0.043	0.837	0.136	0.059	0.809
	F	1.003	0.456	0.500	17.336	16.443	**0.000**	0.011	0.005	0.945	0.469	0.203	0.653
	W	1.225	0.557	0.456	0.003	0.003	0.959	0.044	0.019	0.891	3.803	1.643	0.201
	A * F	2.336	1.062	0.303	10.336	9.804	**0.002**	4.444	1.890	0.170	3.025	1.307	0.254
	A * W	0.025	0.011	0.915	5.625	5.335	**0.021**	1.344	0.572	0.45	0.336	0.145	0.703
	F * W	0.136	0.062	0.804	3.403	3.228	0.073	0.011	0.005	0.945	0.003	0.001	0.972
	A * F * W	1.469	0.668	0.414	1.225	1.162	0.282	1.600	0.680	0.410	0.136	0.059	0.809
**Anger set**	A	0.178	0.077	0.781	0.069	0.033	0.857	8.711	8.308	**0.004**	0.069	0.031	0.861
	F	0.178	0.077	0.781	0.803	0.378	0.539	6.400	6.104	**0.014**	0.336	0.149	0.699
	W	0.178	0.077	0.781	1.003	0.472	0.493	5.878	5.606	**0.018**	0.003	0.001	0.972
	A * F	0.178	0.077	0.781	2.336	1.099	0.295	4.011	3.826	0.051	1.003	0.445	0.505
	A * W	0.711	0.310	0.578	3.025	1.424	0.234	8.711	8.308	**0.004**	4.669	2.074	0.151
	F * W	0.400	0.174	0.677	1.225	0.576	0.448	0.178	0.170	0.681	0.069	0.031	0.861
	A * F * W	1.600	0.697	0.404	0.469	0.221	0.639	2.500	2.384	0.123	0.336	0.149	0.699
**Surprise set**	A	0.711	0.317	0.574	0.178	0.080	0.778	0.178	0.080	0.778	3.520	2.583	0.109
	F	0.044	0.020	0.888	1.344	0.604	0.438	1.344	0.604	0.438	12.247	8.984	**0.003**
	W	0.011	0.005	0.944	0.011	0.005	0.944	0.011	0.005	0.944	1.547	1.135	0.287
	A * F	0.178	0.079	0.779	1.878	0.843	0.359	1.878	0.843	0.359	2.916	2.139	0.144
	A * W	0.011	0.005	0.944	2.500	1.123	0.290	2.500	1.123	0.290	0.007	0.005	0.942
	F * Wa	0.278	0.124	0.725	0.044	0.020	0.888	0.044	0.020	0.888	2.916	2.139	0.144
	A * F * W	0.278	0.124	0.725	0.044	0.020	0.888	0.044	0.02	0.888	0.016	0.012	0.914

Bold *p*-values indicate that the *p*-value is less than 0.05, and bold and underlined *p*-values indicate that the *p*-value is less than 0.01.

**TABLE 4:** Statistical results for the 3-way ANOVA under happiness, sadness, anger, and surprise emotions. The results display scores for animacy, and likability, along with Mean-square, F-values and *p*-values. A represents amplitude, F represents frequency, and W represents waveform.

		Animacy score			Likability score		
		MS	F	p	MS	F	p
**Happiness Set**	A	8.659	9.230	**0.003**	10.000	10.749	**0.001**
	F	7.852	8.370	**0.004**	16.384	17.610	**0.000**
	W	10.967	11.690	**0.001**	16.044	17.245	**0.000**
	A * F	1.667	1.777	0.183	1.547	1.663	0.198
	A * W	0.170	0.182	0.670	0.374	0.402	0.527
	F * W	0.457	0.488	0.485	0.036	0.039	0.844
	A * F * W	0.201	0.214	0.644	0.044	0.048	0.827
**Sadness set**	A	5.878	6.316	**0.012**	11.449	11.318	**0.001**
	F	6.944	7.462	**0.007**	15.129	14.956	**0.000**
	W	4.594	4.936	**0.027**	12.027	11.890	**0.001**
	A * F	1.385	1.489	0.223	2.533	2.505	0.114
	A * W	1.225	1.316	0.252	0.961	0.950	0.330
	F * W	0.025	0.027	0.870	0.729	0.721	0.396
	A * F * W	0.031	0.033	0.856	0.625	0.618	0.432
**Anger set**	A	9.291	10.437	**0.001**	11.808	12.500	**0.000**
	F	4.632	5.203	**0.023**	18.86	19.965	**0.000**
	W	7.754	8.710	**0.003**	12.996	13.757	**0.000**
	A * F	0.819	0.920	0.338	2.304	2.439	0.119
	A * W	0.557	0.626	0.429	0.235	0.249	0.618
	F * W	0.667	0.750	0.387	0.400	0.423	0.516
	A * F * W	0.170	0.191	0.662	0.002	0.002	0.965
**Surprise set**	A	7.415	7.567	**0.006**	8.649	9.357	**0.002**
	F	2.612	2.666	0.103	11.307	12.233	**0.001**
	W	7.511	7.665	**0.006**	11.307	12.233	**0.001**
	A * F	1.225	1.250	0.264	0.529	0.572	0.450
	A * W	0.934	0.953	0.330	0.560	0.606	0.437
	F * W	0.001	0.001	0.972	0.413	0.447	0.504
	A * F * W	0.003	0.003	0.958	0.121	0.131	0.718

Bold *p*-values indicate that the *p*-value is less than 0.05, and bold and underlined *p*-values indicate that the *p*-value is less than 0.01.

**TABLE 5:** The *post hoc* test comparisons results under happiness emotions display mean, *p*-values and Cohen’s d values for emotional scores, animacy scores, and likability scores.

		Factor	Conditions	Mean	p	d
**Happiness Set**	**Happiness Score**	Amp	High	4.228	**0.022**	0.242
			Low	4.000		
		Freq	High	4.044	0.165	0.147
			Low	4.183		
		Wave	Square	4.139	0.617	0.053
			Sine	4.089		
	**Sadness Score**	Amp	High	3.072	1.000	0.000
			Low	3.072		
		Freq	High	2.928	0.061	0.198
			Low	3.217		
		Wave	Square	2.944	0.097	0.175
			Sine	3.200		
	**Anger Score**	Amp	High	3.089	0.445	0.081
			Low	3.200		
		Freq	High	3.056	0.222	0.129
			Low	3.233		
		Wave	Square	3.083	0.401	0.089
			Sine	3.206		
	**Surprise Score**	Amp	High	3.044	0.828	0.023
			Low	3.011		
		Freq	High	3.039	0.885	0.015
			Low	3.017		
		Wave	Square	3.039	0.885	0.015
			Sine	3.017		
	**Animacy Score**	Amp	High	3.680	**0.003**	0.313
			Low	3.369		
		Freq	High	3.672	**0.005**	0.298
			Low	3.377		
		Wave	Square	3.699	**0.001**	0.353
			Sine	3.350		
	**Likability Score**	Amp	High	3.363	**0.002**	0.332
			Low	3.697		
		Freq	High	3.317	**0.000**	0.428
			Low	3.743		
		Wave	Square	3.319	**0.000**	0.423
			Sine	3.741		

Bold *p*-values indicate that the *p*-value is less than 0.05, and bold and underlined *p*-values indicate that the *p*-value is less than 0.01.

**TABLE 6:** The *post hoc* test comparisons results under sadness emotions display mean, *p*-values and Cohen’s d values for emotional scores, animacy scores, and likability scores.

		Factor	Conditions	Mean	p	d
**Sadness Set**	**Happiness Score**	Amp	High	3.000	0.695	0.041
			Low	3.061		
		Freq	High	2.978	0.498	0.072
			Low	3.083		
		Wave	Square	2.972	0.454	0.079
			Sine	3.089		
	**Sadness Score**	Amp	High	3.844	**0.027**	0.234
			Low	4.094		
		Freq	High	3.750	**0.000**	0.417
			Low	4.189		
		Wave	Square	3.967	0.961	0.005
			Sine	3.972		
	**Anger Score**	Amp	High	3.061	0.961	0.005
			Low	3.028		
		Freq	High	3.039	0.836	0.022
			Low	3.050		
		Wave	Square	3.033	0.945	0.007
			Sine	3.056		
	**Surprise Score**	Amp	High	2.950	0.890	0.015
			Low	2.989		
		Freq	High	2.933	0.808	0.026
			Low	3.006		
		Wave	Square	2.867	0.651	0.048
			Sine	3.072		
	**Animacy Score**	Amp	High	3.638	**0.014**	0.262
			Low	3.382		
		Freq	High	3.649	**0.007**	0.285
			Low	3.371		
		Wave	Square	3.623	**0.029**	0.231
			Sine	3.397		
	**Likability Score**	Amp	High	3.324	**0.001**	0.343
			Low	3.681		
		Freq	High	3.298	**0.000**	0.396
			Low	3.708		
		Wave	Square	3.320	**0.001**	0.351
			Sine	3.686		

Bold *p*-values indicate values less than 0.05, and values that are both bold and underlined indicate *p*-values less than 0.01.

**TABLE 7:** The *post hoc* test comparisons results under anger emotions display mean, *p*-values and Cohen’s d values for emotional scores, animacy scores, and likability scores.

		Factor	Conditions	Mean	p	d
**Anger Set**	**Happy Score**	Amp	High	3.078	0.779	0.030
			Low	3.033		
		Freq	High	3.033	0.779	0.030
			Low	3.708		
		Wave	Square	3.078	0.779	0.030
			Sine	3.033		
	**Sadness Score**	Amp	High	3.028	0.856	0.019
			Low	3.000		
		Freq	High	2.967	0.538	0.065
			Low	3.061		
		Wave	Square	2.961	0.491	0.073
			Sine	3.067		
	**Anger Score**	Amp	High	3.806	**0.005**	0.296
			Low	4.117		
		Freq	High	3.828	**0.017**	0.253
			Low	4.094		
		Wave	Square	4.089	**0.022**	0.242
			Sine	3.833		
	**Surprise Score**	Amp	High	3.028	0.860	0.019
			Low	3.000		
		Freq	High	2.983	0.698	0.041
			Low	3.044		
		Wave	Square	3.017	0.972	0.004
			Sine	3.011		
	**Animacy Score**	Amp	High	3.692	**0.002**	0.336
			Low	3.370		
		Freq	High	3.644	**0.026**	0.235
			Low	3.418		
		Wave	Square	3.678	**0.004**	0.306
			Sine	3.384		
	**Likability Score**	Amp	High	3.344	**0.001**	0.358
			Low	3.707		
		Freq	High	3.297	**0.000**	0.456
			Low	3.754		
		Wave	Square	3.336	**0.000**	0.376
			Sine	3.716		

Bold *p*-values indicate values less than 0.05, and values that are both bold and underlined indicate *p*-values less than 0.01.

**TABLE 8:** The *post hoc* test comparisons results under surprise emotions display mean, *p*-values and Cohen’s d values for emotional scores, animacy scores, and likability scores.

		Factor	Conditions	Mean	p	d
**Surprise Set**	**Happiness Score**	Amp	High	3.044	0.571	0.060
			Low	2.956		
		Freq	High	3.011	0.887	0.015
			Low	2.989		
		Wave	Square	3.006	0.944	0.007
			Sine	2.994		
	**Sadness Score**	Amp	High	3.000	0.777	0.027
			Low	3.039		
		Freq	High	2.933	0.435	0.119
			Low	3.106		
		Wave	Square	2.983	0.943	0.050
			Sine	3.056		
	**Anger Score**	Amp	High	3.000	0.777	0.030
			Low	2.956		
		Freq	High	3.039	0.435	0.082
			Low	2.917		
		Wave	Square	2.972	0.943	0.007
			Sine	2.983		
	**Surprise Score**	Amp	High	3.663	0.113	0.167
			Low	3.861		
		Freq	High	3.578	**0.003**	0.315
			Low	3.947		
		Wave	Square	3.828	0.294	0.111
			Sine	3.697		
	**Animacy Score**	Amp	High	3.668	**0.007**	0.287
			Low	3.381		
		Freq	High	3.609	0.109	0.169
			Low	3.439		
		Wave	Square	3.669	**0.006**	0.289
			Sine	3.380		
	**Likability Score**	Amp	High	3.342	**0.003**	0.314
			Low	3.652		
		Freq	High	3.320	**0.001**	0.360
			Low	3.674		
		Wave	Square	3.320	**0.001**	0.360
			Sine	3.674		

Bold *p*-values indicate values less than 0.05, and values that are both bold and underlined indicate *p*-values less than 0.01.

In the happiness emotion, amplitude and the interaction of amplitude and frequency showed statistically significant effects. The performance of amplitude under happiness emotion (F = 5.306, *p* < 0.05) and the interaction with frequency (F = 6.392, *p* < 0.05) indicate that they have a significant effect on emotional expression. In addition, animacy (F = 9.230, *p* < 0.01) and likability (F = 10.749, *p* < 0.01) scores also showed significant differences in amplitude, indicating the importance of amplitude in these emotional expressions. Separate effects of frequency were also significant on animacy (F = 8.370, *p* < 0.01) and likability (F = 17.610, *p* < 0.01), pointing to frequency as another key factor influencing the perception of these emotions. The waveform also showed strong effects on animacy (F = 11.690, *p* < 0.01) and likability (F = 17.245, *p* < 0.01).

In the sadness emotion, frequency had a strong and consistent effect on emotional expression (F = 16.443, *p* < 0.01). The analysis on animacy (F = 7.462, *p* < 0.01) and likability (F = 14.956, *p* < 0.01) further confirmed this. This suggests that frequency is critical in mediating the expression of sad emotions. At the same time, amplitude is also significant when expressing sad emotions (F = 5.335, *p* < 0.05), and the interaction between amplitude and waveform has an equally significant impact on the feeling of sadness (F = 5.335, *p* < 0.05). The results indicate that in the context of sad emotions, not only frequency is an important factor, but the specific combination of amplitude and waveform also affects the expression and perception of individual emotions.

In the anger emotion, amplitude (F = 8.308, *p* < 0.01), frequency (F = 6.104, *p* < 0.05), waveform (F = 5.606, *p* < 0.05), amplitude and waveform (F = 8.308, *p* < 0.01) The interaction effects all had statistically significant effects on the expression of anger emotions. Furthermore, amplitude also showed significant effects on animacy (F = 10.437, *p* < 0.01) and likability (F = 12.500, *p* < 0.01). frequency shows an extremely significant impact on the expression of likability (F = 19.965, *p* < 0.01), and the impact on animacy is also significant (F = 5.203, *p* < 0.05). The waveform also showed an extremely significant impact on the expression of likability (F = 13.757, *p* < 0.01) and also had a significant impact on animacy (F = 8.710, *p* < 0.01). These findings reveal that emotional communication under angry emotions is not only fully affected by amplitude, frequency, and waveform.

In the surprise emotion, although the impact of amplitude, frequency, and waveform on the emotion of surprise alone is not significant, their effects on animacy and likability are significant. The effect of amplitude on animacy (F = 7.567, *p* < 0.01) and likability (F = 9.357, *p* < 0.01) was significant. The effect of frequency on likability is very significant (F = 12.233, *p* < 0.01). The effect of the waveform on animacy (F = 7.665, *p* < 0.01) and likability (F = 12.233, *p* < 0.01) was significant.

The results of *post hoc* test comparisons further elucidated the specific role of these factors in different emotional expressions.

In the happiness emotion, there is a significant difference between the high and low amplitudes. Compared with the low amplitude, the high amplitude condition (MD = −0.228, SE = 0.099; *p* < 0.05, d = 0.242) can significantly affect the expression of emotion. In addition, high amplitude also significantly enhanced the perception of animacy (MD = −0.310, SE = 0.104; *p* < 0.01, d = 0.313). In terms of frequency, compared with low frequency, the perception of animacy was higher under high frequency conditions (MD = −0.295, SE = 0.105; *p* < 0.01, d = 0.298). Comparison of waveforms revealed that square waves have a more significant effect than sine waves in enhancing animacy perception (MD = 0.349, SE = 0.104; *p* < 0.01, d = 0.353). In terms of likability, the likability perception under the low amplitude condition was significantly higher (MD = 0.333, SE = 0.106; *p* < 0.01, d = 0.332), and the likability perception under the low frequency condition was higher than that of the high frequency condition (MD = −0.427, SE = 0.105; *p* < 0.01, d = 0.428). The sine waveform is more effective in improving the perception of likability (MD = 0.422, SE = 0.105; *p* < 0.01, d = 0.423).

In the sadness emotion, the perception of sadness emotion in the low amplitude condition was significantly stronger than that in the high amplitude condition (MD = 0.250, SE = 0.113; *p* < 0.05, d = 0.234). Compared with high frequency, the perception of sadness emotion was more significant under low frequency conditions (MD = 0.439, SE = 0.111; *p* < 0.01, d = 0.417). In terms of animacy perception, high amplitude significantly improved animacy perception compared with low amplitude (MD = 0.256, SE = 0.103; *p* < 0.05, d = 0.262), and animacy perception under high frequency conditions was significantly higher than that at low frequency (MD = 0.278, SE = 0.103; *p* < 0.01, d = 0.285). In addition, the square waveform performed better than the sine waveform in improving animacy perception (MD = 0.226, SE = 0.103; *p* < 0.05, d = 0.231). In terms of likability perception, the likability perception under the low amplitude condition was significantly higher than that under the high amplitude condition (MD = 0.357, SE = 0.110; *p* < 0.01, d = 0.343), and the likability perception under the low frequency condition was also significant. high (MD = 0.410, SE = 0.109; *p* < 0.01, d = 0.396). The sine wave was more significant in improving the perception of likability (MD = 0.366, SE = 0.110; *p* < 0.01, d = 0.351).

In the anger emotion, research shows that amplitude, frequency, and waveform have significant effects on emotion perception. Specifically, the low amplitude condition showed a significant increase in the perception of anger emotion compared to the high amplitude condition (MD = 0.311, SE = 0.111; *p* < 0.01, d = 0.296). The perception of anger emotion was stronger in the low-frequency condition than in the high-frequency condition (MD = 0.267, SE = 0.111; *p* < 0.05, d = 0.253). Compared with sine waves, square waves can significantly enhance the perception of anger emotions (MD = −0.256, SE = 0.111; *p* < 0.05, effect size = 0.242). Regarding animacy perception, the high amplitude condition significantly improved animacy perception compared with the low amplitude condition (MD = 0.321, SE = 0.101; *p* < 0.01, d = 0.336). Perception of animacy in the high-frequency condition exceeded that of the low-frequency condition (MD = −0.227, SE = 0.102; *p* < 0.05, d = 0.235). Comparison of square and sine waveforms also showed significant differences (MD = 0.294, SE = 0.101; *p* < 0.01, d = 0.306), demonstrating the effectiveness of square waveforms in improving animacy perception. In terms of likability perception, the likability perception in the low amplitude condition was significantly higher (MD = 0.362, SE = 0.107; *p* < 0.01, d = 0.358). The perception of likability in the low-frequency condition was significantly higher than that in the high-frequency condition (MD = 0.458, SE = 0.106; *p* < 0.01, d = 0.456). Sine waveforms had a significantly better effect on likability perceptions than square waves (MD = 0.380, SE = 0.107; *p* < 0.01, d = 0.375).

In the surprise emotion, the difference between high frequency and low frequency was significant (MD = 0.369, SE = 0.123; *p* < 0.01, d = 0.315), and the perception of surprise emotion was more obvious under the low frequency condition. In terms of animacy perception, the difference between the high and low amplitude conditions was also significant (MD = −0.287, SE = 0.105; *p* < 0.01, d = 0.287), with increased perceived animacy in the high amplitude condition. The effect of changes in waveform on animacy perception is also significant (MD = −0.289, SE = 0.105; *p* < 0.01, d = 0.289), and square waves can enhance animacy perception. In terms of likability perception, likability perception was enhanced in the low amplitude condition (MD = 0.310, SE = 0.104; *p* < 0.01, d = 0.314). Likability perceptions were stronger in the low frequency condition (MD = 0.354, SE = 0.104; *p* < 0.01, d = 0.360). The sine waveform was more effective in enhancing the perception of likability (MD = 0.354, SE = 0.104; *p* < 0.01, d = 0.360).

## 6 Discussion

In this section, we validate our hypothesis through experimental results and compare our findings with existing research on human-computer interaction. We also discuss the limitations of our research method and provide a general direction for future research.

### 6.1 Hypothesis testing

#### 6.1.1 Major effects

Firstly, we test our hypothesis. Our results show that the combination of a mechanical tail and bionic eyeball improves the accuracy, animacy, and likability of emotional expression compared to using the bionic eyeball alone, supporting H1. The results of experiment 2 show significant improvement in emotional expression, animacy, and likability compared to experiment 1. This suggests that combining tail and eyes in the emotional expression strategy can effectively convey the energy relationship of emotions, the robot’s animacy, and user acceptance. The developed tail movement strategy can assist the robot in expressing emotions better when combined with eyes, resulting in a significant improvement. In Experiment 2, we obtained diverse experimental results by controlling three factors: amplitude, frequencies, and motion waveform of the mechanical tail. When discussing amplitude, high amplitude improves the expression of happy and angry emotions, while low amplitude improves the expression of sad emotions. This is consistent with the energy magnitude defined in emotion theory, where the amplitude of the tail can be used to express the energy relationship of emotions and improve the accuracy of emotional expression. However, unlike H2, amplitude does not significantly affect emotional expression under surprise emotion. This may be because surprise emotion prioritizes frequency performance over amplitude perception in space. Therefore, the difference between high and low frequencies is significant under surprise emotion. When discussing frequencies, it has been found that low frequency significantly improves the expression of sad, angry, and surprised emotions. This is consistent with the energy magnitude defined in emotion theory, which suggests that the frequency of the tail can be used to express the energy relationship of emotions and improve the accuracy of emotional expression. However, unlike H3, frequencies did not have a significant effect on emotional expression in a happy mood, and no significant effect of high frequency on emotion was observed. Happiness may be more closely linked to amplitude than spatial frequency perception. Therefore, in a happy mood, the difference between high and low amplitudes is significant. The reason why the influence of high frequency on emotion is not significant may be due to the limited emotional samples, and the tail with high frequency may not be well coordinated with the emotional movements of the eyes in the study to express emotions. The perception of the high-frequency tail may change too rapidly in space, which may result in insufficient attention being paid to the high-frequency tail to significantly affect the emotional expression of the robot. In our exploration of the impact of waveform on emotional expression, we observed that utilizing a square wave significantly enhanced the robot’s ability to express anger. Conversely, the effect of waveform on the expression of happiness, sadness, and surprise was not significant, indicating that the influence of waveform on different emotions’ expression is inconsistent and does not fully meet the expectations set by the definition of energy magnitude in emotion theory. Specifically, changes in the current waveform only affected acceleration and did not gain significant effects in curvature, so the waveform of the tail did not significantly improve the accuracy of emotional expression in describing the energy relationship of emotions, which contradicts our original H4. This discrepancy may stem from the use of square and sine waves as waveform variables, where the visual perception changes of the tail were not pronounced enough to highlight the significance of waveform in the robot’s emotional expression. Additionally, the mechanical reciprocating motion using a fixed waveform might have diluted participants’ perception of the movement trajectory, thereby diminishing the contribution of waveform to the effectiveness of emotional expression.

#### 6.1.2 Interaction effects

The study found that for a single factor, high amplitude, high frequency, and square wave performed better in expressing animacy, while low amplitude, low frequency, and sine performed better in expressing likability. This suggests that the amplitude, frequencies, and waveform of the tail can be used as influencing factors to improve the robot’s performance in terms of animacy and user acceptance. The frequency factor does not significantly affect the animacy factor under surprise emotion. This may be because the expression of animacy under surprise emotion is more focused on amplitude perception and trajectory perception in space. Generally, the animacy of the robot is more related to large and fast regular limb movements in space, while likability is more related to small and slow smooth limb movements in space. However, for the multi-factor case, the amplitude, frequency and waveform of the mechanical tail do not significantly affect the expression of animacy and affection when they interact bidirectionally. These findings support H5 and H6, indicating that the two-way interaction of any two factors cannot be used to express the animacy and popularity of the robot. When two individual factors are combined, they may not produce an obvious correlation effect, resulting in subjects perceiving insufficient differences in visual effects. This establishes the impression of the two-way interaction on the animacy and likability of the robot. However, the right combination of frequency and amplitude can enhance the expressive power of the action to convey a specific emotion. The investigation elucidated that the dynamics of frequency and amplitude exhibit a synergistic relationship, significantly influencing the bidirectional interactions governing the motion of the cat-like robot’s tail. Nevertheless, the anticipated tripartite interaction among waveform, frequencies, and amplitude did not manifest with statistical significance. This absence of a discernible, consistent interaction across the three variables diverges from the predictions set forth in H7. Such an inconsistency may underscore the complexities inherent in integrating these factors to predictably influence emotional expression, animacy, and likability in robotic entities. The difficulty in establishing a definitive correlation between waveform, frequencies, amplitude, and their cumulative effect on the robot’s capacity for emotional expression indicates the necessity for a more refined and detailed categorization and analysis of these variables. This nuanced approach could potentially illuminate the subtle mechanics underpining the multifaceted nature of robotic emotional expression, thereby offering deeper insights into the optimization of robotic design for enhanced human-robot interaction. Additionally, our findings suggest that the waveform has a minimal effect on the expression of emotion, animacy, and likability. Furthermore, the bidirectional interaction between frequency and amplitude emphasizes their potential synergistic effect. Future research should aim to separate the mechanism of the waveform and explore it further.

### 6.2 Comparison with previous studies

This study successfully expressed the energy relationship of emotions through the combination of a mechanical tail and a bionic eyeball. The tail improved the expression of four emotions: happiness, sadness, anger and surprise. The study addressed the issue of emotional expression not being obvious in happy and angry emotions when using a single eye model, as observed in the relevant literature on emotional expression of eyes (Jatmiko, Ginalih, and Darmakusuma, 2020). This study demonstrates that tail movement strategies can assist a robot in producing more credible emotional output. The tail’s frequency, amplitude, and waveform can express the energy of emotions to varying degrees, which is consistent with previous literature on physical emotional expression (Lee et al., 2014). However, unlike previous experiments, this study uses the tail as the carrier. The article thoroughly discusses the collaboration between the three factors and the eye expression, and the interaction between the parameters. The effect of these parameters on the cat-like robot provides a valuable reference for the further development of similar robots.

### 6.3 Limitations of the study

A limitation of this study is that it simply analyzed a small number of waveforms and emotional types. This may explain why the interaction of three factors did not significantly improve emotional expression. In order to reveal more complex results, additional types of waveforms and emotions should be included. Additionally, participants were exposed to each robot action video once, amounting to multiple viewings. This repeated exposure may induce visual fatigue towards the robot’s behavior, potentially leading to varied evaluations of emotions over time. To enhance the human-computer interaction experience, future studies could consider adding different types of waveforms and emotions, and inviting experimental participants to experience the robot offline.

## 7 Conclusion

This paper investigates how to utilize the strategy of tail movement to enhance the emotional expression of a cat-like robot. The inclusion of a mechanical tail altered the robot’s emotional expression, as evidenced by a comparison of emotional scores before and after its addition. The combination of a mechanical tail and bionic eyeballs not only improved the accuracy of emotional expression, but also the animacy and likability of the expression, compared to using only bionic eyeballs. The auxiliary effects of frequencies, amplitude and different waveforms on emotion expression were analyzed by three-way ANOVA. The results revealed that for emotional expression, frequency and amplitude exhibited varying degrees of influence on emotional expression when considered individually. However, it was observed that waveforms solely exhibited a significant impact on the expression of anger emotion. For animacy and likability, the frequency, amplitude and waveform of the mechanical tail swing can significantly contribute to their enhancement when acting separately. Nevertheless, the interaction between any two factors has no significant effect on these attributes. Moreover, the experimental results of the interaction of the three factors are not universal and consistent, which means that they can neither significantly enhance the expression of emotion nor show stronger animacy and likability. Future research could explore how different waveforms, emotional types, acceleration, and curvature impact emotional expression in interactive robots, building upon previous findings (Saerbeck and Bartneck, 2010). This study introduces a mechanical tail to augment the emotional expression of robots across various emotional states, building upon prior research. The investigation validates the supplementary impact of the mechanical tail on emotional expression under distinct parameters. Through comparative analysis, this study discerns particular conditions conducive to enhancing emotional expression, thereby contributing to the improvement of interaction experiences in future human-robot interactions.

## Data Availability

The raw data supporting the conclusions of this article will be made available by the authors, without undue reservation.
